# The regions within the N-terminus critical for human glucagon like peptide-1 receptor (hGLP-1R) cell Surface expression

**DOI:** 10.1038/srep07410

**Published:** 2014-12-15

**Authors:** Aiysha Thompson, Venkateswarlu Kanamarlapudi

**Affiliations:** 1 Institute of Life Science 1, College of Medicine, Swansea University, Singleton Park, Swansea, SA2 8PP, UK

## Abstract

The hGLP-1R is a target for the treatment of type 2 diabetes and belongs to the class B family of GPCRs. Like other class B GPCRs, the GLP-1R contains an N-terminal signal peptide (SP) and undergoes *N*-linked glycosylation, which are important for its trafficking and maturation. This study analysed the role of the SP, the hydrophobic region after the SP (HRASP), glycosylation and the conserved residues within the N-terminus in GLP-1R trafficking. HGLP-1R targeted to the cell surface showed no SP, and the SP deleted mutant, but not the mutants defective in SP cleavage, showed cell surface expression, demonstrating the importance of SP cleavage for hGLP-1R cell surface expression. The N-terminal deletions of hGLP-1R revealed that the HRASP, not the SP, is essential for cell surface expression of GLP-1R. Further, inhibition of hGLP-1R glycosylation prevented cell surface expression of the receptor. Mutation of Trp^39^, Tyr^69^ and Tyr^88^, which are required for agonist binding, in the GLP-1R abolished cell surface expression of the receptor independent of the SP cleavage or *N*-linked glycosylation. In conclusion, the N-terminus of hGLP-1R regulates receptor trafficking and maturation. Therefore this study provides insight into the role of hGLP-1R N-terminus on the receptor cell surface expression.

Glucagon like peptide-1 (GLP-1) is a polypeptide hormone secreted by the intestinal L-cells into the blood in response to food intake^1^,^2^,^3^. It is an effective insulinotropic agent, which lowers blood glucose levels and increases insulin secretion^1^,^4^,^5^. It acts as an agonist to the GLP-1 receptor (GLP-1R), a family B G-protein coupled receptor (GPCR). The binding of GLP-1 to the GLP-1R results in insulin secretion from pancreatic β-cells, making human GLP-1R (hGLP-1R) an important target in the treatment of type 2 diabetes^1^,^6^.

The family B GPCRs contain a N-terminal domain signal peptide (SP) sequence that is often critical for the synthesis and processing of the receptor^7^. The SP is about 20 amino acids (aa) long and contains a run of hydrophobic residues. The first stage of protein targeting, during its synthesis, is insertion into the endoplasmic reticulum (ER) by binding to the signal recognition particle (SRP), which is usually mediated by the SP^8^. For example, deleting the SP sequence of the thyrotropin receptor (TR) abolished its functionality^9^,^10^. However, the SP of the corticotropin-releasing factor (CRF) type 2a receptor, although present, is incapable of mediating ER targeting^11^,^12^. Further, the SP of the CRF_1_ receptor is required for its expression but not for its function^13^. The GLP-1R contains a cleavable N-terminal SP (23 aa long), its cleavage was not required for synthesis of the receptor but was essential for cell surface expression of the receptor^14^. Mutation of the SP (Ala^21^Arg) to prevent its cleavage has been shown to result in retention of the GLP-1R within the ER. Further, a mutation of Glu^34^ was shown to facilitate GLP-1R cell surface expression when the SP was deleted^14^. The aa sequence following the SP in the GLP-1R, Gly^27^-Trp^39^, is relatively hydrophobic and it has previously been suggested that this region may be recognised by the SRP for synthesis of the receptor^14^,^15^.

GPCRs synthesised in the ER translocate to the Golgi before being targeted to the cell surface. In this process, GPCRs undergo post- or co-translational modifications including glycosylation, methylation, phosphorylation, sulfation and lipid addition^16^,^17^. The *N*-linked glycosylated GPCRs are processed further in the ER and Golgi before translocation and insertion into the plasma membrane^18^. The GLP-1R has been shown to undergo *N*-linked glycosylation at positions Asn^63^, Asn^82^ and Asn^115^ within the ER^19^,^20^.

The hGLP-1R has three residues, Trp^39^, Tyr^69^ and Tyr^88^, within its N-terminal domain that are important for agonist binding^21^,^22^,^23^. Trp^39^ has importance in maintaining the structure of the N-terminal domain of the GLP-1R by interacting with Tyr^42^, Phe^66^ and the adjacent disulphide bond (Cys^46^-Cys^71^)^24^. It has been demonstrated that GLP-1 could not bind and activate the GLP-1R when Trp^39^ was substituted with Ala or Phe^21^. Further, Phe^22^, Ile^23^ and Leu^26^ of GLP-1 interacts with Trp^39^ in addition to Val^36^, Asp^67^, Tyr^69^, Arg^121^ and Leu^123^ of the GLP-1R^22^. Tyr^69^, which is centrally located within the N-terminal domain, interacts with Asp^67^ and has been shown to be involved in GLP-1 binding to its receptor^23^. Tyr^88^ is involved in making the hydrophobic agonist binding site, which interacts with Leu^32^ of GLP-1 and Leu^26^ of Exendin-4^22^,^23^. Although, Trp^39^, Tyr^69^ and Tyr^88^ residues within the GLP-1R have been shown to be required for agonist binding, their role in hGLP-1R trafficking, function and *N*-linked glycosylation are currently unknown.

The GLP-1R is a major therapeutic target in the treatment of type 2 diabetes, therefore a better understanding of its membrane trafficking is of high importance. This study determined that the SP is cleaved in the mature hGLP-1R. Cell surface expression was almost abolished with a mutation of the SP (A21R) to prevent its cleavage, demonstrating that the cleavage of the SP was essential for cell surface expression of the hGLP-1R. Although the role of the SP in family B GPCR trafficking is well established, the significance of the hydrophobic region after the SP (HRASP) is unclear. Here, the HRASP was shown to be necessary for efficient hGLP-1R trafficking to the cell surface. Further, this study indicated that the hGLP-1R undergoes *N*-linked glycosylation and only the mature fully glycosylated form is found at the cell surface. It was also demonstrated that preventing cleavage of the SP inhibited hGLP-1R cell surface expression by affecting *N*-linked glycosylation. Additionally, mutating Trp^39^, Tyr^69^ and Tyr^88^ within the hGLP-1R abolished cell surface expression of the receptor without affecting *N*-linked glycosylation and cleavage of the SP.

## Results

### HGLP-1R expressing at the cell surface shows no SP

**Figure 1:**
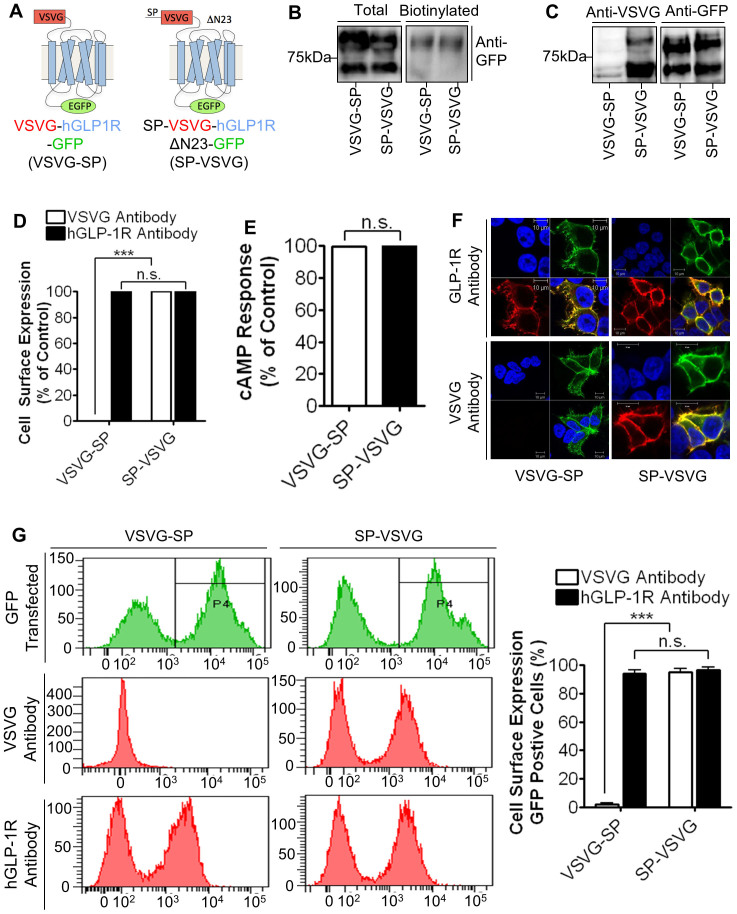
HGLP-1R expressing at the cell surface shows no SP. (A) HEK293 cells transfected with SP-VSVG and VSVG-SP constructs. (B) Total and cell surface biotinylated hGLP-1R expression was assessed by immunoblotting using the anti-GFP antibody. (C) Total hGLP-1R expression was assessed by immunoblotting using the anti-VSVG and anti-GFP antibodies. (D) Cell surface expression was assessed by ELISA using the anti-VSVG and anti-hGLP-1R antibodies. (E) Agonist stimulated cAMP production was measured to assess hGLP-1R activity. (F) Immunofluorescence showing cell surface expression of hGLP-1R, EGFP (green) and the anti-hGLP-1R antibody (red) overlay shown in yellow and nuclear staining with DAPI in blue. (G) Cells surface expression of hGLP-1R constructs assessed by flow cytometry. Data are mean ± SEM, n = 3, n.s. p > 0.05; *** p < 0.001.

It has been shown previously that the mature hGLP-1R expressing at the cell surface is without the SP (1–23 aa)^14^. To confirm whether the SP is cleaved off from the mature hGLP-1R that is targeted to the plasma membrane, constructs containing a GFP-epitope at the C-terminus and VSVG-epitope at the N-terminus before (SP-VSVG) or after the SP (VSVG-SP) were generated (Figure 1A). HEK293 cells transfected with these constructs were analysed for hGLP-1R cell surface expression by ELISA (Figure 1D), immunofluorescence (Figure 1F) and flow cytometry (Figure 1G) using the anti-hGLP-1R and anti-VSVG antibodies. HEK293 cells expressing the SP-VSVG construct showed cell surface expression of the receptor with both antibodies. However, HEK293 cells expressing the VSVG-SP construct showed signal at the cell surface with the anti-hGLP-1R antibody but not with the VSVG antibody (100 ± 0.58% versus 0 ± 0.58% by ELISA and 93.8 ± 2.61% versus 1.83 ± 1.07% by flow cytometry with the anti-hGLP-1R antibody [p < 0.001] versus the anti-VSVG antibody [p > 0.05], respectively). This result suggested that the SP is cleaved in the membrane targeted hGLP-1R.

Both the SP-VSVG and VSVG-SP constructs showed a doublet (~65 kDa and ~85 kDa in size) when the lysates of HEK293 cells transfected with these constructs were immunoblotted with the anti-GFP antibody (Figure 1C). In addition, the SP-VSVG but not the VSVG-SP construct showed a doublet in the immunoblot probed with the anti-VSVG antibody, indicating that the SP is cleaved off from the hGLP-1R before it is targeted to the cell surface. Further, when HEK293 cells expressing these constructs were subjected to cell surface biotinylation, only a single band at ~85 kDa was observed in the total lysate (Figure 1B). This demonstrated the ~85 kDa band represents the mature form of the hGLP-1R that targeted to the cell surface.

The GLP-1R is a Gαs coupled GPCR and therefore the activity of the receptor was assessed by measuring cAMP produced in hGLP-1R expressing cells stimulated with agonist (Figure 1E). The VSVG-SP construct had 99.57 ± 0.43% (p > 0.05) cAMP accumulation compared to the SP-VSVG construct, confirming the VSVG-SP is functionally no different from the SP-VSVG construct. Furthermore, the cAMP producing activity of SP-VSVG (which contains both VSVG and GFP tags) is similar to that of the hGLP-1R with no tag or either of the VSVG-tag or GFP-tag, indicating that the attachment of the VSVG and GFP tags to the hGLP-1R had no effect on the activity of the receptor (Figure S1). For further experimentation, the SP-VSVG construct was used as the wild type (WT) control.

### Cleavage of the SP is necessary for targeting the hGLP-1R to the cell surface

**Figure 2:**
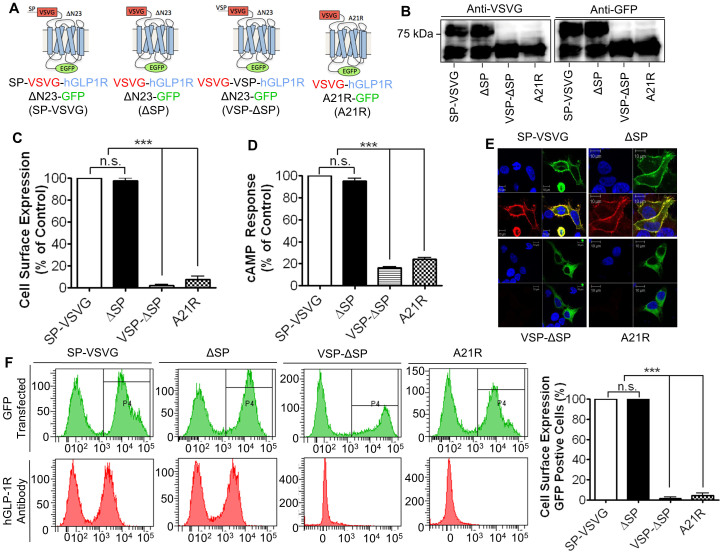
Cleavage of the SP is required for hGLP-1R cell surface expression. (A) HEK293 cells transfected with the indicated hGLP-1R constructs. (B) Total hGLP-1R expression was assessed by immunoblotting using the anti-VSVG and anti-GFP antibodies. (C) Cell surface expression was assessed by ELISA using the anti-hGLP-1R antibody. (D) Agonist stimulated cAMP production was measured to assess hGLP-1R activity. (E) Immunofluorescence showing cell surface expression of hGLP-1R, EGFP (green) and the anti-hGLP-1R antibody (red) overlay shown in yellow and nuclear staining with DAPI in blue. (F) Cells surface expression of hGLP-1R constructs was analysed by flow cytometry. Data are mean ± SEM, n = 3, n.s. p > 0.05; *** p < 0.001.

Next, the importance of the SP cleavage in hGLP-1R cell surface expression was determined. The cell surface expression of the hGLP-1R without the SP (ΔSP), the hGLP-1R containing the SP replaced with viral SP (VSP-ΔSP) and the hGLP-1R defective in cleaving the SP (A21R) was compared to the SP-VSVG WT control (Figure 2A). HEK293 cells transfected with these constructs were analysed for their effect on hGLP-1R cell surface expression (assessed by ELISA [Figure 2C], immunofluorescence [Figure 2E] and flow cytometry [Figure 2F] using the anti-hGLP-1R antibody) and activity (assessed by cAMP [Figure 2D]). The ΔSP construct showed cell surface expression (assessed by ELISA [97.43 ± 2.57%, p > 0.05], immunofluorescence and flow cytometry [100 ± 0.58%, p > 0.05]) similar to that of the SP-VSVG WT control. Additionally, the ΔSP construct showed 95.24 ± 2.55% (p > 0.05) agonist induced cAMP production, confirming the hGLP-1R without the SP is functionally similar to the control hGLP-1R. In contrast, VSP-ΔSP and A21R constructs showed very little cell surface expression (2.33 ± 0.64% and 7.82 ± 2.73% by ELISA, and 1.9 ± 1.7% and 4.40 ± 2.21% by flow cytometry, p < 0.001, respectively), which was confirmed by immunofluorescence. The cAMP activity of the VSP-ΔSP and A21R constructs in agonist stimulated cells was also low (16.18 ± 1.25% and 24.09 ± 1.47%, p < 0.001, respectively). Immunoblotting of the cell lysates expressing the above mentioned constructs suggested that the SP of VSP-ΔSP and A21R was not cleaved and as a result produced a single band at the lower molecular weight of ~65 kDa with both the anti-GFP and anti-VSVG antibodies (Figure 2B), confirming the expression of an immature receptor. This result demonstrated that the SP is specific to the hGLP-1R and mutating this sequence prevents cleavage of the SP and thereby targeting of the hGLP-1R to the cell surface.

### The sequence after the SP is required for hGLP-1R cell surface expression

**Figure 3:**
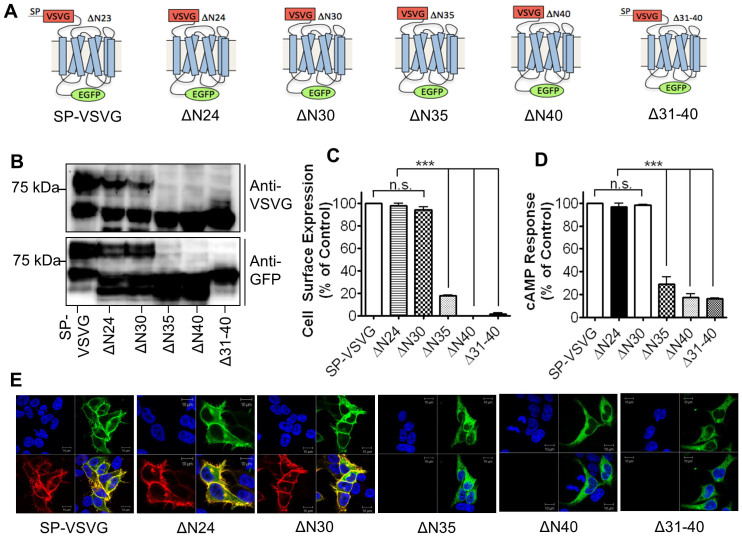
The sequence after the SP is essential for hGLP-1R cell surface expression. (A) HEK293 cells were transfected with the indicated N-terminal deleted constructs. (B) Total hGLP-1R expression was assessed by immunoblotting using the anti-VSVG and anti-GFP antibodies. (C) Cell surface expression was assessed by ELISA using the anti-hGLP-1R antibody. (D) Agonist stimulated cAMP production was measured to assess hGLP-1R activity. (E) Immunofluorescence showing cell surface expression of hGLP-1R, EGFP (green) and the anti-hGLP-1R antibody (red) overlay shown in yellow and nuclear staining with DAPI in blue. Data are mean ± SEM, n = 3, n.s p > 0.05; *** p < 0.001.

A number of deletions were made within the HRASP of the hGLP-1R and analysed for their effect on the cell surface expression and activity of the receptor (Figure 3A). For this purpose, the cell surface expression of the N-terminal deleted hGLP-1R mutants in HEK293 cells was analysed by ELISA (Figure 3C). Removal of either 24 aa (ΔN24) or 30 aa (ΔN30) from the N-terminal domain had no effect on hGLP-1R cell surface expression (98.15 ± 2.12% and 94.39 ± 2.65%, p > 0.05, respectively). However, deleting 35 aa (ΔN35) from the N-terminus significantly reduced hGLP-1R cell surface expression and deleting 40 aa (ΔN40) abolished the cell surface expression altogether (17.8 ± 0.61% and 0.21 ± 0.16, p < 0.001, respectively). These results were also confirmed by immunofluorescence (Figure 3E). Additionally, the cAMP production of the receptor in agonist stimulated cells reflected cell surface expression of the receptor (Figure 3D). Agonist induced cAMP production of the ΔN24 and ΔN30 mutants (96.73 ± 3.28% and 98.21 ± 0.89%, p > 0.05, respectively) were similar to that produced by the WT. In contrast, hGLP-1R activity was significantly reduced when either 35 aa (ΔN35) or 40 aa (ΔN40) were deleted from the N-terminal domain (28.82 ± 6.32% and 17.53 ± 2.99%, p < 0.001, respectively). Consequently, the region between 31–40 aa was deleted (Δ31–40) from the hGLP-1R and analysed for the deletions effect on hGLP-1R cell surface expression and cAMP production. Cell surface expression (1.2 ± 1.32%, p < 0.001) and cAMP production (16.36 ± 0.17%, p < 0.001) of the hGLP-1R were almost abolished in the Δ31–40 mutant when compared to that of the WT, indicating the importance of this region in trafficking the receptor to the cell surface. Immunofluorescence confirmed these results and showed hGLP-1R expression to be intracellular. Immunoblotting confirmed that the reduced cell surface expression of these deletion mutants was not due to alterations in their expression levels (Figure 3B).

### *N*-linked glycosylation is essential for hGLP-1R cell surface expression

**Figure 4:**
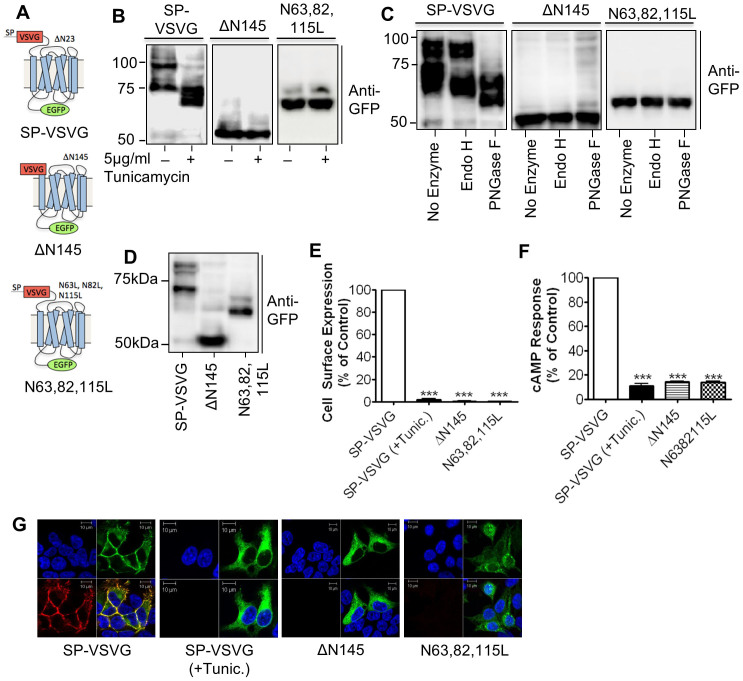
*N*-linked glycosylation is essential for hGLP-1R cell surface expression. (A) HEK293 cells were transfected with either SP-VSVG, ΔN145 or N63, 82, 115L plasmid DNA. (B) Cells were treated without or with 5 μg/ml tunicamycin for 48 h. The cells were lysed and the cell lysates were immunoblotted with the anti-GFP antibody. (C) Post nuclear supernatant fractions of HEK293 cells were treated with either no enzyme, Endo H or PNGase F for 60 min at 37°C and immunoblotted with the anti-GFP antibody. (D) Total hGLP-1R expression was assessed by immunoblotting using the anti-GFP antibody. (E) Cell surface expression was assessed by ELISA using the anti-hGLP-1R antibody. (F) Agonist stimulated cAMP production was measured to assess hGLP-1R activity. (G) Immunofluorescence showing cell surface expression of hGLP-1R, EGFP (green) and the anti-hGLP-1R antibody (red) overlay shown in yellow and nuclear staining with DAPI in blue. Data are mean ± SEM, n = 3, *** p < 0.001.

The hGLP-1R has been shown to be *N*-linked glycosylated at positions Asn^63^, Asn^82^ and Asn^115^ within the ER^19^,^20^. Therefore, HEK293 cells transfected with either the WT SP-VSVG, ΔN145 or N63, 82, 115L constructs (Figure 4A) were used to assess the importance of *N*-linked glycosylation in hGLP-1R cell surface expression. Immunoblotting of the SP-VSVG WT control showed the doublet at ~65 kDa and ~85 kDa (Figure 4B). Treatment of SP-VSVG with a *N*-linked glycosylation inhibitor, tunicamycin, shifted this doublet to ~60 kDa and 65 kDa. This shift is used as a readout assay to assess hGLP-1R *N*-linked glycosylation and showed that the hGLP-1R is *N*-linked glycosylated. The hGLP-1R with the *N*-terminal domain removed (ΔN145) showed only a single band at ~50 kDa in immunoblotting. As the glycosylation sites were removed in the ΔN145 mutant, no change in mobility was seen when treated with tunicamycin. Additionally, the N63, 82, 115L mutant, with all three *N*-linked glycosylation sites mutated, of the hGLP-1R showed a single band at ~60 kDa, which was also unaltered by treatment with tunicamycin.

HGLP-1R glycosylation can be removed by treatment with both PNGase F and Endo H, indicating the receptor is *N*-linked glycosylated^25^. PNGase F cleaves oligomannoses and both hybrid and complex N-glycans whereas Endo H cleaves oligomannoses and some hybrid glycans. Therefore, the WT SP-VSVG, ΔN145 or N63, 82, 115L constructs were digested with Endo H or PNGase F enzymes and analysed for their band pattern by immunoblotting (Figure 4C). Treatment of the SP-VSVG WT control lysate with Endo H caused a shift in the lower band mobility only from ~65 kDa to ~60 kDa. However, treatment with PNGase F shifted both bands to ~60 kDa and 65 kDa, which mimicked the effect of tunicamycin and thereby confirmed that the hGLP-1R is *N*-linked glycosylated by oligomannoses and both hybrid and complex *N*-glycans in the mature form. In contrast, the lysates of HEK293 cells expressing either the ΔN145 or N63, 82, 115L mutants showed no shift in band pattern when treated with either Endo H or PNGase F, confirming that they are not glycosylated.

The deleted (ΔN145) and mutated (N63, 82, 115L) hGLP-1R constructs were used to assess the importance of *N*-linked glycosylation for cell surface expression of the receptor by ELISA (Figure 4E) and immunofluorescence (Figure 4G). HGLP-1R cell surface expression was abolished in both mutations when compared to the WT (0.48 ± 0.48% and 0.14 ± 0.07%, p < 0.001, respectively). Further, when cell expressing the SP-VSVG control construct was treated with tunicamycin, cell surface expression was abolished (1.94 ± 0.64%, p < 0.001). This was confirmed further by immunofluorescence where cell surface expression was seen for the SP-VSVG construct with good colocalisation between GFP-tag and cell surface staining with the anti-hGLP-1R antibody. However, the ΔN145 and N63, 82, 115L mutants and the SP-VSVG construct treated with tunicamycin only showed intracellular expression of GFP and no cell surface expression with the anti-hGLP-1R antibody. Immunoblotting demonstrated that the reduction in cell surface expression of the mutants was not a result of reduced protein expression (Figure 4D). Consistent with the reduced cell surface expression, the ΔN145 and N63, 82, 115L mutants and the SP-VSVG construct treated with tunicamycin caused reduced cAMP production in agonist stimulated cells (14.25 ± 0.29%, 13.61 ± 0.93% and 11.08 ± 1.61%, p < 0.001, respectively, Figure 4F). Therefore, preventing hGLP-1R glycosylation by either deleting the N-terminal domain or mutating the glycosylation sites within the N-terminal domain drastically reduced cell surface expression of the receptor.

### Effect of point mutations within the N-terminal domain on the cell surface expression of the hGLP-1R

**Figure 5:**
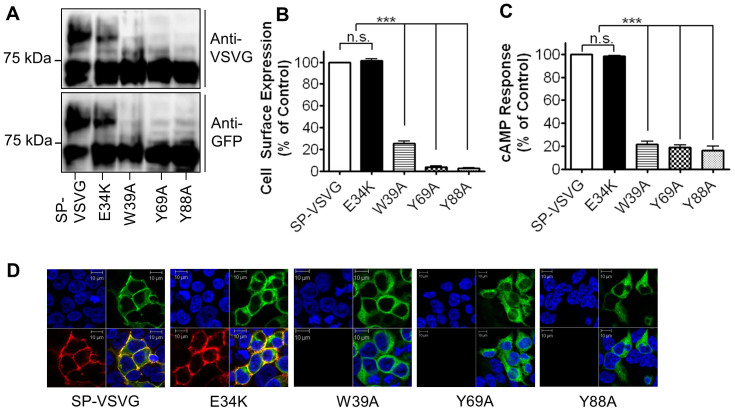
The effect of various point mutations within the N-terminal domain of hGLP-1R on the cell surface expression of the receptor. HEK293 cells were transfected with the indicated N-terminal mutated constructs. (A) Total hGLP-1R expression was assessed by immunoblotting using the anti-VSVG and anti-GFP antibodies. (B) Cell surface expression was assessed by ELISA using the anti-hGLP-1R antibody. (C) Agonist stimulated cAMP production was measured to assess hGLP-1R activity. (D) Immunofluorescence showing cell surface expression of hGLP-1R, EGFP (green) and the anti-hGLP-1R antibody (red) overlay shown in yellow and nuclear staining with DAPI in blue. Data are mean ± SEM, n = 3, n.s p > 0.05; *** p < 0.001.

A number of N-terminal residues conserved across the family B GPCRs were mutated within the hGLP-1R to assess their effect on cell surface expression of the receptor (estimated by ELISA [Figure 5B] and immunofluorescence [Figure 5D]) and activity (assessed by cAMP accumulation [Figure 5C]). The total protein expression of the mutants was determined by immunoblotting using both the anti-GFP and anti-VSVG antibodies (Figure 5A). Substitution of the negatively charged Glu^34^ with a positively charged Lys residue (E34K) had no significant effect on cell surface expression (101.6 ± 1.59%, p > 0.05) or activity (98.45 ± 0.26%, p > 0.05) of the receptor. Total protein expression levels of the E34K mutant were similar to that of the SP-VSVG control construct. The W39A mutation significantly reduced hGLP-1R cell surface expression (25.12 ± 2.43%, p < 0.001) and agonist stimulated cAMP production (21.72 ± 2.4%, p < 0.001). Additionally, the Y69A mutant of the hGLP-1R showed very low cell surface expression (3.74 ± 0.8%, p < 0.001) and reduced agonist induced cAMP production (18.91 ± 2.3%, p < 0.001). Further, the Y88A mutation within the N-terminal domain of the hGLP-1R almost abolished cell surface expression of the receptor (2.30 ± 1.05%, p < 0.001) and showed an even further reduction in cAMP production (16.44 ± 3.65%, p < 0.001). Immunoblot analysis confirmed that the reduction in cell surface expression of these mutants was not due to alterations in the mutants protein expression. Consistent with the reduction in the cell surface expression and cAMP producing activity, only a single band was seen at ~65 kDa for these three mutations, indicating the immature receptor. Immunofluorescence also supported the ELISA results as intracellular expression was seen with GFP but no cell surface staining was observed with the anti-hGLP-1R antibody.

### Effect of SP, HRASP and conserved residue mutants on hGLP-1R *N*-linked glycosylation

**Figure 6:**
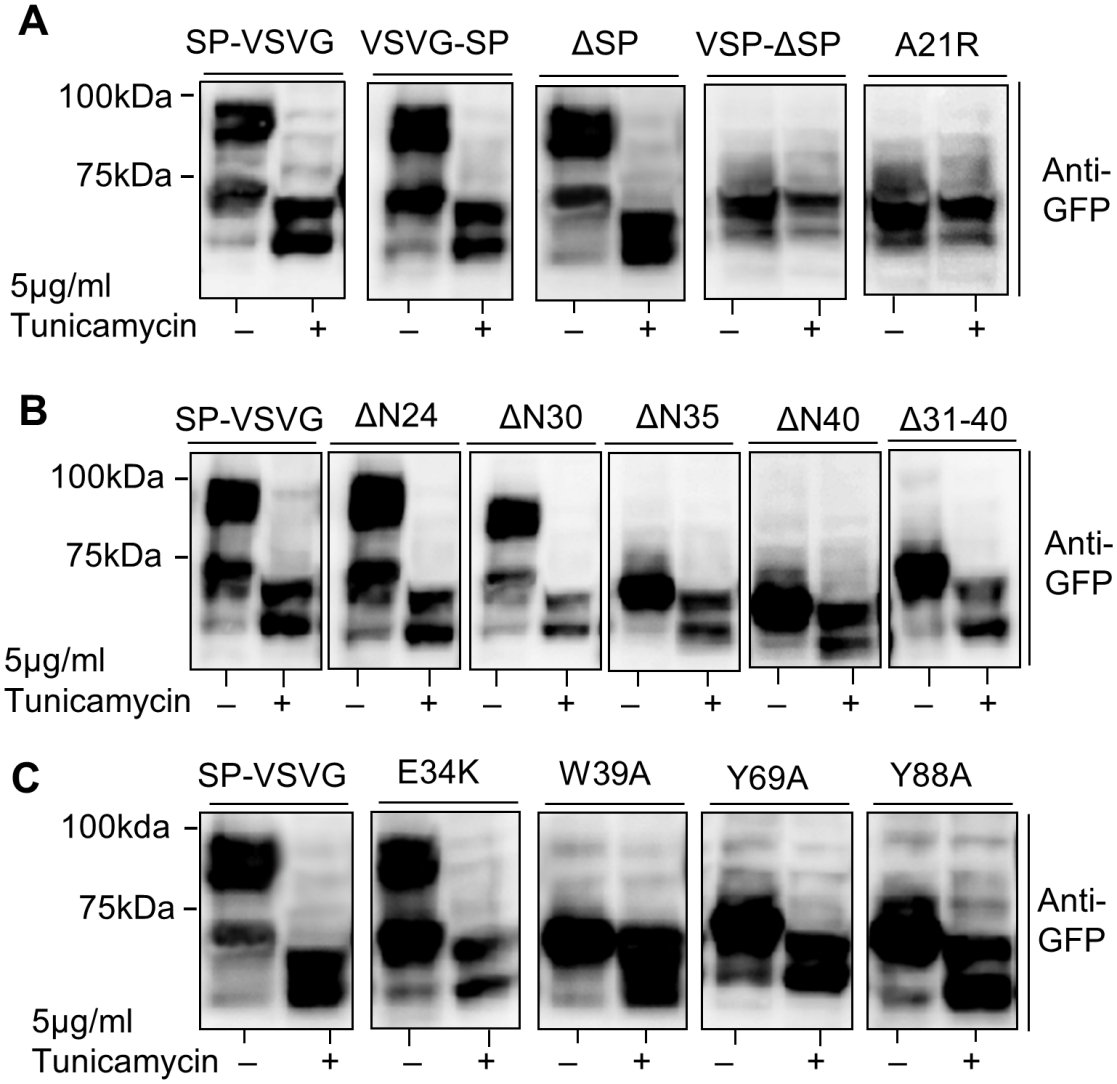
The effect of the SP, HRASP and conserved residue mutations on hGLP-1R glycosylation. HEK293 cells transfected with SP (A), HRASP (B) or the conserved residue (C) mutant constructs treated without or with 5 μg/ml tunicamycin for 48 h. The cells were lysed and the cell lysates were immunoblotted with the anti-GFP antibody.

The importance of the SP, the HRASP and conserved residues (Glu^34^, Trp^39^, Tyr^69^ and Tyr^88^) within the hGLP-1R N-terminus on its *N*-linked glycosylation was determined. For this purpose, cells expressing the constructs were treated without or with tunicamycin and the cell lysates analysed by immunoblotting using the anti-GFP antibody. Like the SP-VSVG WT control construct, the SP deleted construct (ΔSP) showed a doublet in immunoblotting and the doublet mobility was altered with tunicamycin treatment. This suggested the ΔSP mutant was *N*-linked glycosylated in the same way as the WT. The hGLP-1R mutants that prevented cleavage of the SP (VSP-ΔSP and A21R) only showed a single band at ~65 kDa and the band mobility was unaltered when treated with tunicamycin, indicating that these mutants were not *N*-linked glycosylated (Figure 6A). This is most likely because the SP prevents access to the *N*-linked glycosylation sites, as it is not cleaved in these mutants. Additionally, the mutants with deletions within the HRASP of the N-terminus (ΔN35, ΔN40 and Δ31–40) showed a single band at ~65 kDa and a shift in the doublet mobility was seen when treated with tunicamycin, which suggests that these mutants are still glycosylated (Figure 6B).

When the W39A, Y69A and Y88A mutants were left untreated with tunicamycin, a single band at ~65 kDa was observed indicating the immature form of the receptor. However, when treated with tunicamycin there was a shift in the doublet mobility to ~60 kDa and 65 kDa demonstrating these mutations still allowed the receptor to be *N*-linked glycosylated (Figure 6C). Additionally, the E34K mutant showed a doublet similar to that of the WT control in immunoblotting and the doublet mobility also altered with tunicamycin treatment. These results suggest that *N*-linked glycosylation of the receptor is unaltered with the E34K mutation.

### The W39A, Y69A and Y88A mutations do not affect cleavage of the SP

**Figure 7:**
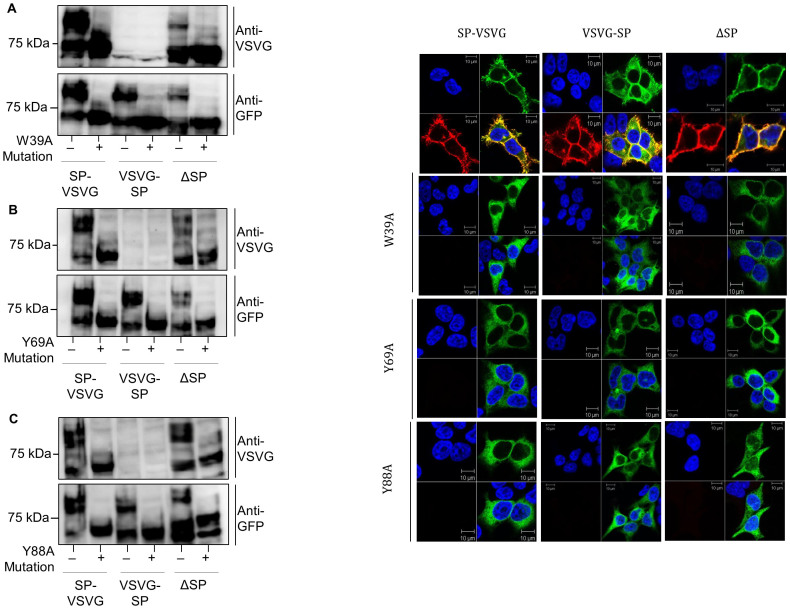
W39A, Y69A and Y88A mutations do not affect cleavage of the SP within the hGLP-1R. (A) Total hGLP-1R expression in HEK293 cells of W39A, Y69A and Y88A mutants in SP-VSVG, VSVG-SP or ΔSP constructs was assessed by immunoblotting using the anti-VSVG and anti-GFP antibodies. (B) Immunofluorescence showing cell surface expression of hGLP-1R, EGFP (green) and the anti-hGLP-1R antibody (red) overlay shown in yellow and nuclear staining with DAPI in blue.

The W39A, Y69A and Y88A mutants in the SP-VSVG, VSVG-SP and ΔSP constructs were used to determine whether these mutations affect cleavage of the SP. The lysates of HEK293 cells expressing these mutants were subjected to immunoblotting with both the anti-GFP and anti-VSVG antibodies to assess total hGLP-1R expression and their effect on its SP cleavage (Figure 7A). The W39A, Y69A and Y88A mutations did not prevent cleavage of the SP when expressed in the SP-VSVG construct. This and expression of these mutants in the ΔSP construct showed expression with both the anti-GFP and anti-VSVG antibodies. However, expression of the VSVG-SP construct with these mutations only showed signal with the anti-GFP antibody but not with the VSVG antibody, suggesting the SP is still cleaved. If the mutations had affected cleavage of the SP, then the mutation would have abolished expression of the VSVG-SP construct and allowed expression of the ΔSP construct at the cell surface. This is because there would be no SP to be cleaved in the ΔSP construct. In immunofluorescence, hGLP-1R cell surface expression was seen with good colocalisation of GFP and the anti-hGLP-1R antibody in all constructs (SP-VSVG, VSVG-SP and ΔSP) without the mutations. However, only intracellular expression was seen with GFP and no cell surface staining with the anti-hGLP-1R antibody for all constructs with the N-terminal mutations (Figure 7B). Taken together, this data suggests that the W39A, Y69A and Y88A mutations did not affect hGLP-1R cell surface expression by preventing cleavage of the SP.

## Discussion

The hGLP-1R construct containing the VSVG-epitope tag at the N-terminal domain before the SP sequence (VSVG-SP) showed signal with the anti-hGLP-1R antibody but not with the anti-VSVG antibody, which indicated that the mature receptor expressed at the cell surface is without its SP. Further, stimulation of cells expressing the VSVG-SP with GLP-1 agonist stimulated cAMP production, confirming that the receptor without the SP is functionally active. These results are in agreement with a previous study, which showed the mature hGLP-1R expressed at the cell surface is without the SP^14^. These findings are also consistent with that of other family B GPCRs including the vasoactive intestinal peptide (VPAC1) receptor^26^ and CRF1 receptor^13^ where the SP is cleaved during synthesis. However, the SP of VPAC1 was found to play a critical role in the receptors targeting as deletion of the SP resulted in the synthesis but prevented trafficking of the receptor to the cell surface. It was suggested that the SP of the VPAC1 receptor is cleaved during trafficking to the plasma membrane, most likely in the ER^26^. Additionally, the SP is of the CRF1 receptor reduced cell surface expression but still retained its functionality^13^. The hGLP-1R with the SP deletion (ΔSP), was shown in this study to function exactly like the receptor with the SP present. This contradicts a previous study, which showed the SP deleted hGLP-1R is synthesised but does not express at the cell surface^14^. The reason for the variation in results is unclear. In this study, the hGLP-1RΔSP was expressed with the VSVG-epitope tag at the N-terminus whereas Huang et al (2010) expressed the same deletion construct with a HA-epitope tag. However, it was observed that the hGLP-1RΔSP without any epitope tag at the N-terminus also targets to the cell surface, indicating that the difference in the N-terminal tag between studies may not be the reason for variation in the results. Within this study, the hGLP-1R showed specificity to its SP sequence because replacing it with the viral SP (VSP-ΔSP) allowed protein synthesis but the cell surface expression was reduced. The A21R mutation (-3 position of the SP cleavage site) allowed synthesis of the hGLP-1R but prevented cleavage of the SP and therefore cell surface expression was reduced, which is consistent with a previous study^14^. Taken together, this study demonstrates that cleavage of the SP is required for hGLP-1R cell surface expression and the SP sequence is specific to the hGLP-1R. This is similar to the specificity demonstrated for the CRF1, as replacement of the CRF1 SP with the CRF2a SP abolished expression of the receptor^11^.

The aa sequence following the SP, Gly^27^-Trp^39^, is relatively hydrophobic (HRASP) and it has previously been suggested that this region may be recognised by the SRP and allow for subsequent synthesis of the receptor^14^,^15^. A similar region within the endothelin B receptor (ETBR), Gln^28^-Trp^34^, was shown to be important in receptor trafficking to cell surface by facilitating translocation across the ER membrane^27^. To examine the role of the HRASP in hGLP-1R trafficking, deletions were made within the HRASP region and assessed for their effect on hGLP-1R cell surface expression. Deleting up to 30 aa of the N-terminal domain of the hGLP-1R had no effect on cell surface expression of the receptor, whereas deletion of up to 40 aa or 31–40 aa abolished hGLP-1R cell surface expression. Therefore, these results suggest that residues 31–40 within the HRASP are important for hGLP-1R cell surface expression and cAMP production. However, the 31–40 aa deletion within the hGLP-1R had no effect on the cleavage of the SP or *N*-linked glycosylation, indicating that the HRASP is not required for either cleavage of the SP or *N*-linked glycosylation of the receptor. It is possible that, like in the ETBR, this region may be important in hGLP-1R translocation across the ER membrane, but requires further studies to confirm this possibility.

The GLP-1R expressed in CCL39 fibroblasts^28^, HEK293^14^ and CHO cells^20^ has previously been shown to produce a two band pattern in immunoblotting, representing different *N*-linked glycosylation states. Consistent with this, the hGLP-1R expressed in HEK293 cells in this study showed a doublet in immunoblotting. Further, treatment with tunicamycin, an *N*-linked glycosylation inhibitor^29^, or deletion of the N-terminus (ΔN145) or mutating the glycosylation sites (N63, 82, 115 L) prevented glycosylation of the hGLP-1R, confirming the hGLP-1R is glycosylated in the N-terminus. Moreover, hGLP-1R glycosylation can be removed by treatment with both PNGase F and Endo H, indicating the receptor is *N*-linked glycosylated. The lysates of cell surface biotinylated hGLP-1R expressing cells showed only the top band of the characteristic two band pattern in immunoblotting, demonstrating it as the fully glycosylated and mature receptor present at the cell surface. This is consistent with a previous study, which showed that only the high molecular weight band of the rat GLP-1R binds the GLP-1 agonist^28^. Taken together, the data in this study confirmed that only the fully glycosylated and mature receptor is found at the cell surface and that mutations and deletions of the glycosylation sites prevented cell surface expression and activity of the receptor. Additionally, tunicamycin inhibited glycosylation of the SP deleted (ΔSP) mutant confirming it also underwent *N*-linked glycosylation. This study demonstrated that preventing cleavage of the SP (A21R or VSP) also inhibits *N*-linked glycosylation, suggesting the SP may prevent access to the glycosylation sites required for hGLP-1R cell surface expression.

In addition to conserved glycosylation sites, the hGLP-1R contains a number of amino acids within the N-terminal domain that are highly conserved among family B GPCRs. A substitution of Glu^34^ to a positively charged residue has previously been shown to partially compensate for the lack of the SP, where no GLP-1R expression was demonstrated^14^. However, in this study the E34K mutation within the hGLP-1R showed no significant effect on the cell surface expression of the receptor. This is expected since the SP deleted (ΔSP) mutant showed no effect on hGLP-1R cell surface expression. It has previously been shown that a mutation of Trp^39^ abolished GLP-1 binding to the GLP-1R, as the imidazole ring structure in this position is important for agonist binding^21^,^23^. In this study, the W39A mutation abolished hGLP-1R cell surface expression, demonstrating that the imidazole ring structure at this position is also required for cell surface expression of the receptor. Tyr^69^ and Tyr^88^ within the hGLP-1R have also been shown to be important in binding to the agonist, Exenatide, but the reason for this was undetermined^22^,^23^. In this study, the Tyr^69^ and Tyr^88^ mutations caused a significant loss in hGLP-1R cell surface expression. The Trp^39^, Tyr^69^ and Tyr^88^ mutants interfered with neither cleavage of the SP nor *N*-linked glycosylation of the receptor and therefore it is unlikely that these mutations had any effect on the stability of the receptor. The exact reason for these mutations affecting hGLP-1R maturation and thereby its cell surface expression is still unclear. However, it is possible that these mutations may affect trafficking of the *N*-linked glycosylated hGLP-1R to the Golgi or interfere with further processing within the ER and Golgi. This is an area requiring further investigation.

**Figure 8:**
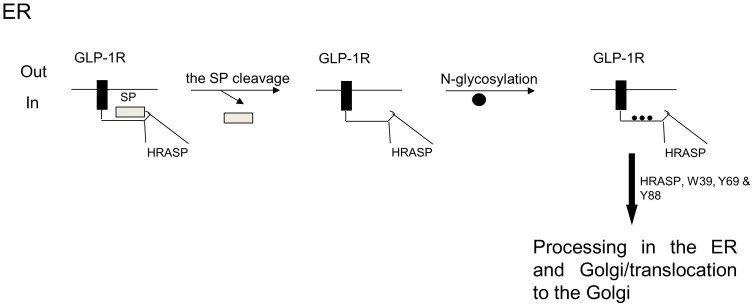
Proposed schematic model of hGLP-1R cell surface expression as deduced from the present study. Within the ER the SP is cleaved to reveal *N*-linked glycosylation sites. The receptor is then glycosylated within the ER and Golgi prior to trafficking to the plasma membrane.

In summary, this study revealed that the SP sequence of the hGLP-1R is cleaved during processing of the receptor. Cleavage of the SP is not essential for hGLP-1R synthesis but is required for glycosylation and trafficking of the receptor to the cell surface. Moreover, the SP is specific to the hGLP-1R. The hGLP-1R is *N*-linked glycosylated and only a fully glycosylated receptor is present at the cell surface. Furthermore, the sequence within the HRASP, 31–40, was found to be critical for hGLP-1R cell surface expression but not for cleavage of the SP or glycosylation of the receptor. The conserved residues, Trp^39^, Tyr^69^ and Tyr^88^, within the N-terminal domain were required for cell surface expression of the hGLP-1R as mutating these residues abolished cell surface expression while not interfering with cleavage of the SP or glycosylation of the receptor. Overall, the results presented in this study suggest that the SP may prevent access to Asn^63^, Asn^82^ and Asn^115^ glycosylation sites within hGLP-1R. With cleavage of the SP, the glycosylation sites are exposed and the receptor undergoes *N*-linked glycosylation. The glycosylated receptor then traffics to the Golgi and then to the plasma membrane. The HRASP (31–40 aa) and Trp^39^, Tyr^69^ and Tyr^88^ residues are critical for hGLP-1R cell surface expressing and most likely play a role in trafficking the receptor from the ER or interfere with further processing within the ER and Golgi (Figure 8).

## Methods

### Antibodies and other reagents

The primary antibodies used were rabbit anti-vesicular stomatitis virus glycoprotein (VSVG) (Immunoblotting, Abcam Biochemicals), mouse anti-VSVG (ELISA and immunofluorescence, Sigma), mouse anti-green fluorescent protein (GFP) (Roche), mouse anti-hGLP-1R (ELISA and immunofluorescence, R&D Systems), mouse anti-hGLP-1R (Immunoblotting, Santa Cruz). The Cy3-conjugated anti-mouse immunoglobulin G (IgG) secondary antibody (Jackson Laboratories) was used for immunofluorescence. The horseradish peroxidase (HRP)-conjugated anti-mouse and anti-rabbit IgG (GE Healthcare) secondary antibodies were used for immunoblotting. Enhanced chemiluminescence (ECL) select reagent was obtained from GE Healthcare. GLP-1 (Liraglutide) was from Novo Nordisk. All other chemicals were from Sigma unless otherwise stated.

### Plasmids

The full-length hGLP-1RΔN23 cDNA was amplified from mammalian gene collection (MGC) clone 142053 (Source Bioscience) by polymerase chain reaction (PCR) using High Fidelity Taq DNA polymerase (Roche Applied Science) and sequence specific primers containing EcoRI restriction site and VSVG-tag coding sequence (5′ primer), and SalI restriction site and no stop codon (3′ primer)^30^. SP-VSVG-hGLP-1RΔN23 cDNA was amplified by overlap PCR using VSVG-hGLP-1RΔN23 cDNA as the template, the sense primer, containing EcoRI restriction site, the SP (1–23 aa) coding sequence followed by VSVG coding sequence and 3′ primer. The cDNA was digested with EcoRI and SalI, and cloned in frame into the same sites of pEGFP-N1 vector (Clontech) for expression as the N-terminus VSVG-tagged (after the SP) and the C-terminus GFP-tagged fusion protein in mammalian cells (SP-VSVG-hGLP-1RΔN23-GFP). The point mutations within the hGLP-1R were generated using Quickchange II XL site-directed mutagenesis kit (Stratagene) and SP-VSVG-hGLP-1RΔN23-GFP plasmid as the template^31^. The mutants with internal deletions (Δ) within the N-terminus of hGLP-1R were generated using Q5 site directed mutagenesis kit (New England Biolabs) and SP-VSVG-hGLP-1RΔN23-GFP plasmid as the template.

### Cell culture and transfection

Human embryonic kidney 293 (HEK293) cells were maintained at 37°C in a 5% CO2 humidified environment in Dulbecco's modified Eagle medium (DMEM; serum free medium [SFM]) supplemented with 10% foetal calf serum, 2 mM glutamine, 100 U/ml penicillin and 0.1 mg/ml streptomycin (full serum medium [FSM]). Cells were transiently transfected for 48 h using JetPrime transfection reagent (Polyplus; 2 μl/μg DNA) according to the manufacturer's instructions.

### Enzyme linked immunosorbent assay (ELISA)

This was carried out as described previously with unpermeabilised cells to quantify cell surface expression^32^. Briefly, HEK293 cells expressing the hGLP-1R were serum starved for 1 h and then stimulated without or with agonist at 37°C/5% CO2. Where indicated, cells were incubated without or with inhibitors for 30 min prior to stimulation with agonist at 37°C/5% CO2. Cells were then fixed with 4% paraformaldehyde (PFA) for 5 min and non-specific binding sites blocked with 1% bovine serum albumin (BSA) made in Tris buffered saline (TBS) (1% BSA/TBS) for 45 min. Cells were incubated with either the anti-hGLP-1R or anti-VSVG mouse antibody (diluted 1:15000) in 1% BSA/TBS for 1 h, washed with TBS and then incubated with the HRP-conjugated anti-mouse IgG (diluted 1:5000) in 1% BSA/TBS for 1 h. Cells were washed and developed using 1-step Ultra TMB-ELISA substrate (Bio-Rad) for 15 min and the reaction stopped by adding an equal volume of 2 M sulphuric acid. The optical density was read at 450 nm using a plate reader.

### Immunofluorescence

Intracellular localisation of hGLP-1R expression was assessed by immunofluorescence as described previously^32^. Briefly, cells were serum starved for 1 h and where indicated cells were pre incubated without or with inhibitors at the indicated concentration for 30 min. Cells were then incubated with either the anti-hGLP-1R or anti-VSVG mouse antibody (diluted 1:5000) in 1% BSA/DMEM for 1 h at 4°C and then stimulated without or with agonist in the presence of inhibitor at 37°C/5% CO2. Cells were then fixed with 4% PFA for 30 min. Cells were permeabilised with 0.2% Triton X-100 made in phosphate buffered saline (PBS) for 10 min, blocked in blocking buffer (1% BSA made in wash buffer [0.1% Triton X-100 in PBS]) for 30 min and then incubated with the Cy3-conjugated anti-mouse antibody (diluted 1:200 in blocking buffer) for 1 h. Cells were then washed 3 times with wash buffer and incubated with DAPI (4′,6-diamidino-2-phenylindole dihydrochloride, 1 mg/ml) diluted 1:2000 in PBS to stain nucleus. Coverslips were mounted on glass microscopic slides using mounting solution (0.1 M Tris-hydrochloric acid [HCl], pH 8.5, 10% Mowiol 50% glycerol) containing 2.5% DABCO (1,4 diazabicyclo (2.2.2) octane). Immunofluorescence staining was visualised using a Zeiss LSM710 confocal microscope fitted with a 63× oil immersion lens.

### cAMP assay

Cells were serum starved for 1 h and then stimulated without or with 100 nM GLP-1 for 1 h at 37°C/5% CO2 in the presence of 0.25 mM phosphodiesterase inhibitor Ro201724. Cells were lysed and cAMP levels in the cell lysates were estimated using the cAMP direct immunoassay kit (Abcam).

### Flow cytometry

Cells in suspension were incubated in blocking buffer (0.2% BSA/PBS) for 1 h at 4°C and then with either the anti-hGLP-1R or anti-VSVG mouse antibodies (diluted 1:100 in blocking buffer) for 1 h at 4°C. Cells were washed 3 times with PBS and incubated with the Cy3-conjugated anti-mouse antibody, diluted 1:100 in blocking buffer for 1 h at 4°C in the dark. Cells were washed 3 times and incubated with 7-aminoactinomycin D (7-AAD) diluted 1:100 in blocking buffer for 5 min at 4°C in the dark. Cells were resuspended in 1 ml fluorescence-activated cell sorting (FACS) buffer (0.2% BSA, 0.05% sodium azide in PBS) and analysed using BD FACs Aria flow cytometer (BD Bioscience) and BD FACS DIVA software.

### Cell lysates

To make cell lysates, HEK293 cells expressing the hGLP-1R were washed 3 times with ice cold PBS and lysed in ice cold modified RIPA lysis buffer (10 mM Tris-HCl, pH 7.5, containing 10 mM ethylenediaminetetraacetic acid [EDTA], 1% nonyl phenoxypolyethoxylethanol [NP40], 0.1% sodium dodecyl sulphate [SDS], 0.5% sodium deoxycholate and 150 mM Sodium Chloride [NaCl]) with 1% mammalian protease inhibitors. Cell lysates were incubated at 4°C for 15 min and then centrifuged at 22000 × g for 10 min at 4°C. The supernatant was collected and ½ volume of 3× SDS-polyacrylamide gel electrophoresis (PAGE) sample loading buffer (75 mM Tris HCl, pH 6.8 containing 3% SDS, 30% glycerol, 0.003% bromophenol blue and 0.3 M dithiothreitol [DTT]) was added and left at room temperature for 1 h. These cell lysates were used to hGLP-1R expression by immunoblotting using the anti-GFP and anti-VSVG antibodies.

### Surface biotinylation

This was performed as described previously^13^. Cells were washed with ice cold PBS containing 1 mM calcium chloride (CaCl2) and 1 mM magnesium chloride (MgCl2) and incubated at 4°C for 1 h with 0.5 mg/ml Sulpho-NHS-LC-Biotin (Thermo Scientific). Cells were then incubated for 10 min at 4°C with 100 mM glycine in TBS to quench any remaining reactive biotin cross linker and lysed in ice cold modified RIPA lysis buffer with 1% mammalian protease inhibitors. Cell lysates were incubated with Streptavidin Magnetic Beads (Invitrogen) at 4°C for 2 h. Beads were washed 3 times with lysis buffer and the bound protein eluted in 1× SDS-PAGE sample loading buffer (25 mM Tris HCl, pH 6.8, containing 1% SDS, 10% glycerol, 0.001% bromophenol blue and 0.1 M dithiothreitol [DTT]). The lysate not incubated with beads was mixed with ½ volume of 3× SDS PAGE sample loading buffer and used to assess total hGLP-1R. Total and biotinylated cell surface receptors were detected by immunoblotting.

### Immunoblotting

Proteins were separated in a SDS-PAGE gel by electrophoresis and transferred onto polyvinylidene fluoride (PDVF) membrane^33^. Membranes were blocked with TBST (TBS with 0.1% tween 20) containing 5% milk powder (blocking buffer) for 1 h at room temperature or overnight at 4°C. Membranes were immunoblotted with the anti-GFP mouse antibody (diluted 1:500 in blocking buffer) for 1 h at room temperature or overnight at 4°C. Membranes were washed and then incubated with the HRP-conjugated anti-mouse secondary antibody (diluted 1:2500 in blocking buffer) for 1 h at room temperature. Membranes were then incubated in ECL select substrate and bands visualised using the ChemiDocTM XRS system (Bio-Rad)^34^,^35^. Blots probed with the anti-GFP mouse antibody were stripped with western blot stripping buffer (Thermo Scientific) and reprobed with the anti-VSVG rabbit antibody (diluted 1:1000) in blocking buffer) and the HRP-conjugated anti-rabbit secondary antibody (diluted 1:2500 in blocking buffer) as described above.

### Tunicamycin treatment

This was carried out as described previously^20^. Briefly, cells were treated with 5 μg/ml tunicamycin at the time of transfection. After 48 h of transfection, cells were lysed and subjected to immunoblotting.

### Glycosidase treatment

This assay was carried out as described previously^14^. Cells harvested from a 10 cm plate by trypsinisation were resuspended in 1 ml homogenisation buffer (10 mM Tris HCl, pH 7.5, 1 mM EDTA, 1 mM phenylmethanesulfonylfluoride [PMSF]) containing 1% mammalian protease inhibitors and incubated on ice for 15 min. Cells were then sonicated at 80% amplitude for 3 × 10 sec with 1 min intervals. The lysate was centrifuged at 300 × g for 10 min at 4°C to pellet nuclei and unbroken cells. An aliquot of post-nuclear supernatant fraction (50 μg of protein) was incubated with glycoprotein denaturing buffer at room temperature for 1 h and then treated without or with 500 units of either PNGase F or Endo H for 1 h at 37°C. Reactions were stopped with the addition of ½ volume of 3× SDS-PAGE sample loading buffer and subjected to immunoblotting.

### Data analysis

Data were analysed using the GraphPad Prism program. All data are presented as means ± standard error of the mean (SEM) of three independent experiments. Statistical comparisons between a control and test value was made by a two-tailed unpaired student t-test. Statistical analysis between multiple groups were determined by the Bonferroni's post test after one-way or two-way analysis of variance (ANOVA), where p > 0.05 was considered as statistically not significant (n.s.), and p < 0.05, p < 0.01 and p < 0.001 shown as *, ** and *** respectively, was considered statistically significant. Concentration response curves were also fitted using Prism, according to a standard logistic equation. Confocal images shown in the figures are representative of 190–200 transfected cells from three different experiments. Similarly, immunoblotting data shown in the figures are representative of three independent experiments.

## Author Contributions

A.T. and V.K. designed research; A.T. and V.K. performed research; A.T. and V.K. analysed data; A.T. and V.K. wrote the paper.

## Supplementary Material

Supplementary Information Suppl Figure 1 legend

Supplementary Information Suppl. Figure 1
