# Supplementary material for: The regions within the N-terminus critical for human glucagon like peptide-1 receptor (hGLP-1R) cell Surface expression
Source: Sci Rep. 2014 Dec 15;4:7410. doi: 10.1038/srep07410 (PMC4344312; doi:10.1038/srep07410)
Supplement: Supplementary Information — Suppl Figure 1 legend [file srep07410-s1.doc]

# The regions within the N-terminus critical for human glucagon like peptide-1 receptor (hGLP-1R) cell Surface expression

Aiysha Thompson and Venkateswarlu Kanamarlapudi

**Figure S1. The effect of epitope tagging on hGLP-1R activity.** (A) Schematic view of VSVG- or GFP- or both tagged hGLP-1R constructs used in this study. (B) HEK293 cells were co-transfected with the indicated hGLP-1R construct and pGL4.29-Luc-CRE reporter plasmid and assessed cAMP production as readout for the hGLP-1R activity. (C) HEK293 cells transfected with the indicated construct were assessed for cell surface expression of hGLP-1R by immunofluorescence. In immunofluorescence, EGFP (green) and GLP-1R antibody (red) overlay was shown in yellow and nuclear staining with DAPI in blue. Data is mean + SEM, n=3.
