# Supplementary figures and images for: The regions within the N-terminus critical for human glucagon like peptide-1 receptor (hGLP-1R) cell Surface expression
Source: Sci Rep. 2014 Dec 15;4:7410. doi: 10.1038/srep07410 (PMC4344312; doi:10.1038/srep07410)

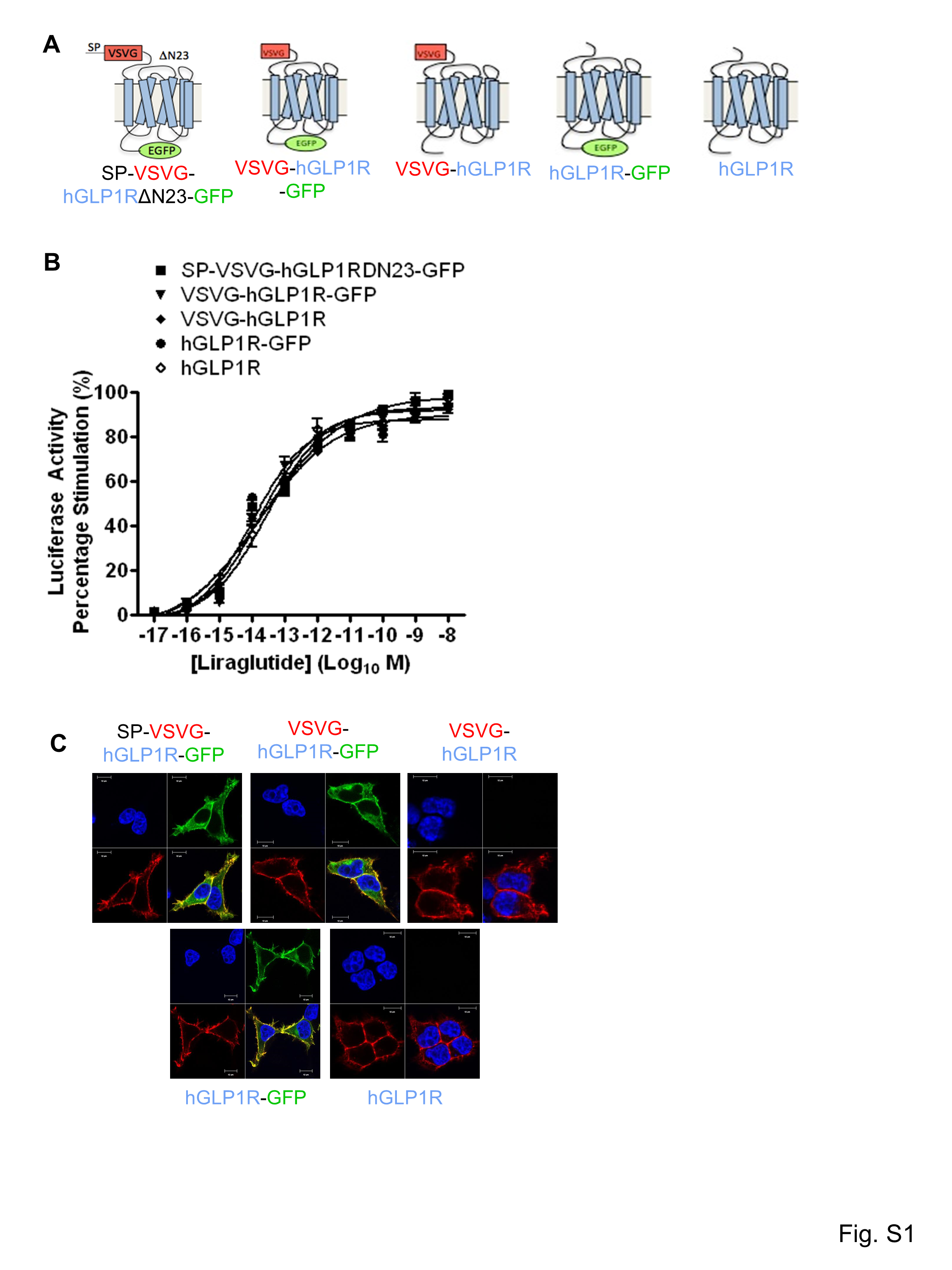

Supplement: Supplementary Information — Suppl. Figure 1 [file srep07410-s2.tiff]
